# Superficial Clear Cell Sarcoma (Melanoma of Soft Parts) of the Large toe in an 80-Year-Old Female With a Rare Cytogenetic Translocation

**DOI:** 10.7759/cureus.11719

**Published:** 2020-11-26

**Authors:** Regina Zambrano, John Moesch, Emily R Davis, Michael R Heaphy, Richard Miller

**Affiliations:** 1 Dermatology, Nova Southeastern University-Kiran C Patel College of Osteopathic Medicine, Fort Lauderdale, USA; 2 Dermatology, Largo Medical Center, Largo, USA; 3 Dermatology, St. Joseph Mercy Livingston, Clinton Township, USA; 4 Dermatopathology, Skin Cancer and Dermatology Institute, Reno, USA; 5 Dermatology, Hospital Corporation of America / University of South Florida Morsani College of Medicine: Largo Medical Center Program, Largo, USA

**Keywords:** clear cell sarcoma, sarcoma, melanoma of soft parts, melanoma, immunohistochemistry, chromosomal translocation, translocation, superficial sarcoma

## Abstract

We present a case of clear cell sarcoma (CCS) on the left large toe of an 80-year-old female. CCS, also known as “melanoma of soft parts,” is a rare soft tissue neoplasm that exhibits melanocytic differentiation. Most cases occur on the distal extremities of young female adults. CCS shares histopathologic and immunohistochemical features with malignant melanoma that cause diagnostic difficulties distinguishing between these entities; therefore, cytogenetic studies of specific translocations are paramount in obtaining the correct diagnosis. The majority of CCS cases reveal a t(12;22)(q13;q12) EWSR1/ATF1 translocation, while a rare subset of CCS demonstrate a t(2;22) (q32:q12) EWS/CREB1 translocation. Our patient presented with a 50-year history of a nodule on the dorsum of her left large toe, with increasing size and tenderness over the past nine months. Histopathology and immunoperoxidase staining indicated CCS as a differential diagnosis. Cytogenetic analysis revealed a translocation in t(2;22) (q32;q12) resulting in the EWSR1/CREB1 gene, confirming a diagnosis of CCS. The translocation, histologic location, and long-standing clinical course exhibited in this case are exceptionally rare, and we hope to inform dermatologists of an uncommon presentation of CCS in an unexpected age group.

## Introduction

Clear cell sarcoma (CCS), also referred to as “melanoma of soft parts,” is a rare, malignant soft tissue neoplasm that mimics the immunohistochemical profile of cutaneous malignant melanoma [1]. CCS accounts for only 1% of soft tissue sarcomas. CCS typically presents as a slow-growing, deep-seated nodular mass that has a predilection for tendons and aponeuroses of the distal extremities on young female adults. Rarer presentations include pigmented lesions or more superficial locations in the epidermis and dermis [2-3]. 

Clear cell sarcoma is most commonly described in pathology as a well-circumscribed proliferation of fusiform cells arranged in nests and fascicles. CCS displays positive immunohistochemical expression of typical melanocytic markers, such as S-100 protein, HMB-45, Melan-A, and MITF, leading many to consider this entity a unique subset of melanoma [3-4]. However, CCS displays translocations of the EWSR1 gene that are genetically distinct from malignant melanoma [2, 5]. The majority of CCS cases detect chromosomal translocation t(12;22)(q13;q12) resulting in a EWSR1/AFT1 fusion transcript, while a minority of cases reveal t(2;22)(q33;q12) translocation, resulting in an uncommon EWSR1/CREB1 fusion transcript [2]. CCS is prone to local recurrence as well as regional and distant metastases many years after primary diagnosis. The five-, 10-, and 20-year disease-specific survival rates have been reported as 67%, 33%, and 10%, respectively [6]. We present a unique case of superficial CCS in an elderly female arising in a lesion that had been present on the left toe for many years.

## Case presentation

**Figure 1:**
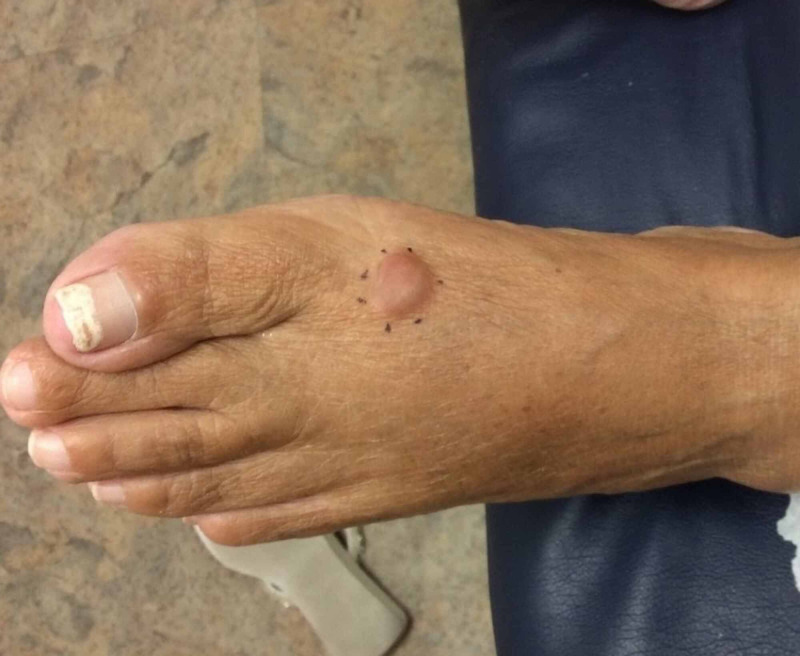
CCS preoperative. 1.4-cm slightly mobile erythematous nodule on the dorsal MTP joint of the left large toe. CCS, clear cell sarcoma; MTP, metatarsopharyngeal

An 80-year-old female with no significant past medical history presented with an enlarging nodule at the base of the left large toe. It had been present for over 50 years as a small, discrete asymptomatic papule. However, over the past nine months, the patient reported a rapid increase in size and tenderness. Upon inspection, the patient was found to have a 1.4-cm erythematous nodule on the dorsal metatarsopharyngeal (MTP) joint of the left large toe (Figure 1). No evidence of lymphadenopathy was identified in the popliteal fossa or inguinal region.

**Figure 2:**
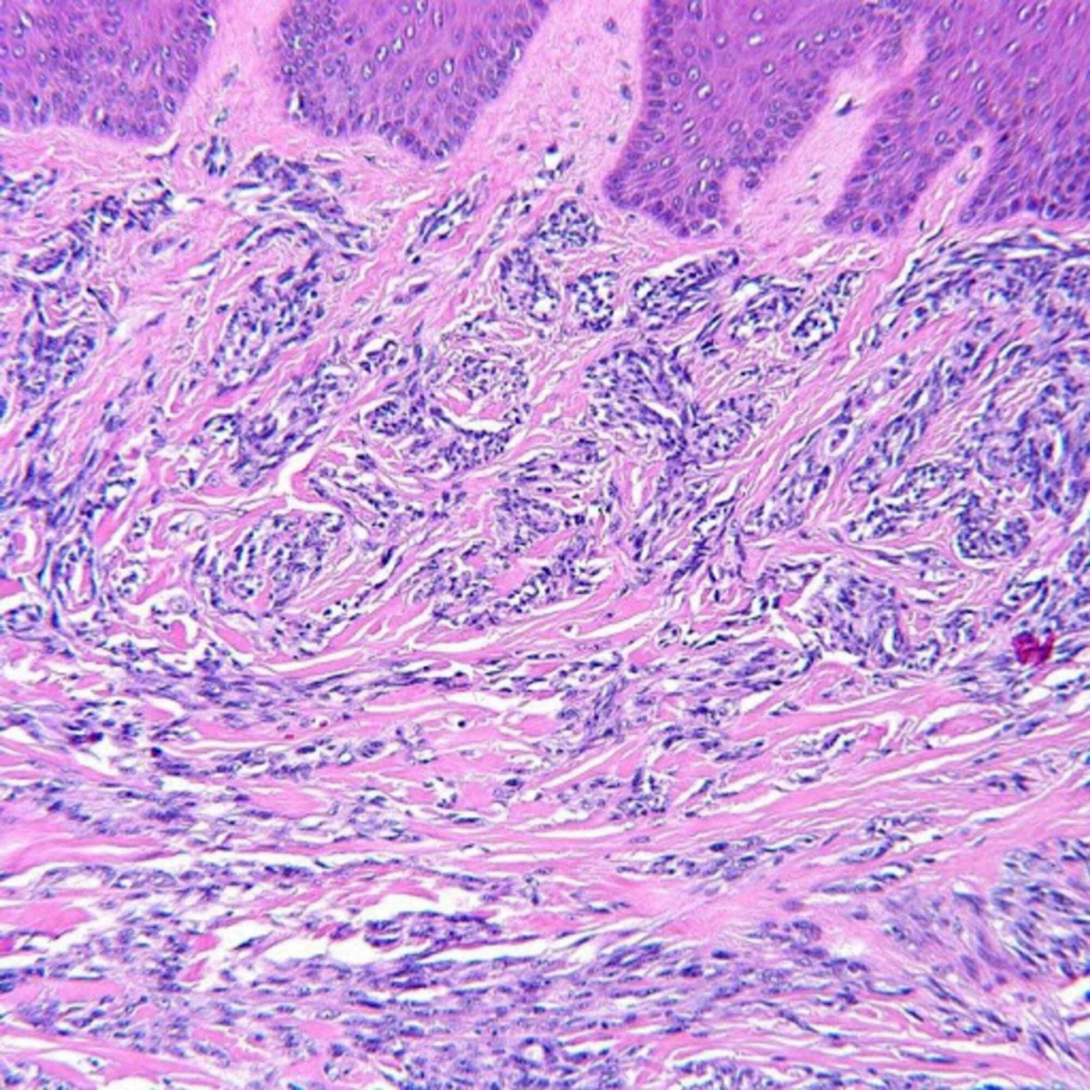
Histopathology. High-power view revealed fusiform and round cells with eosinophilic to clear cytoplasm within the dermis and subcutis (H&E, original magnification x200).

**Figure 3:**
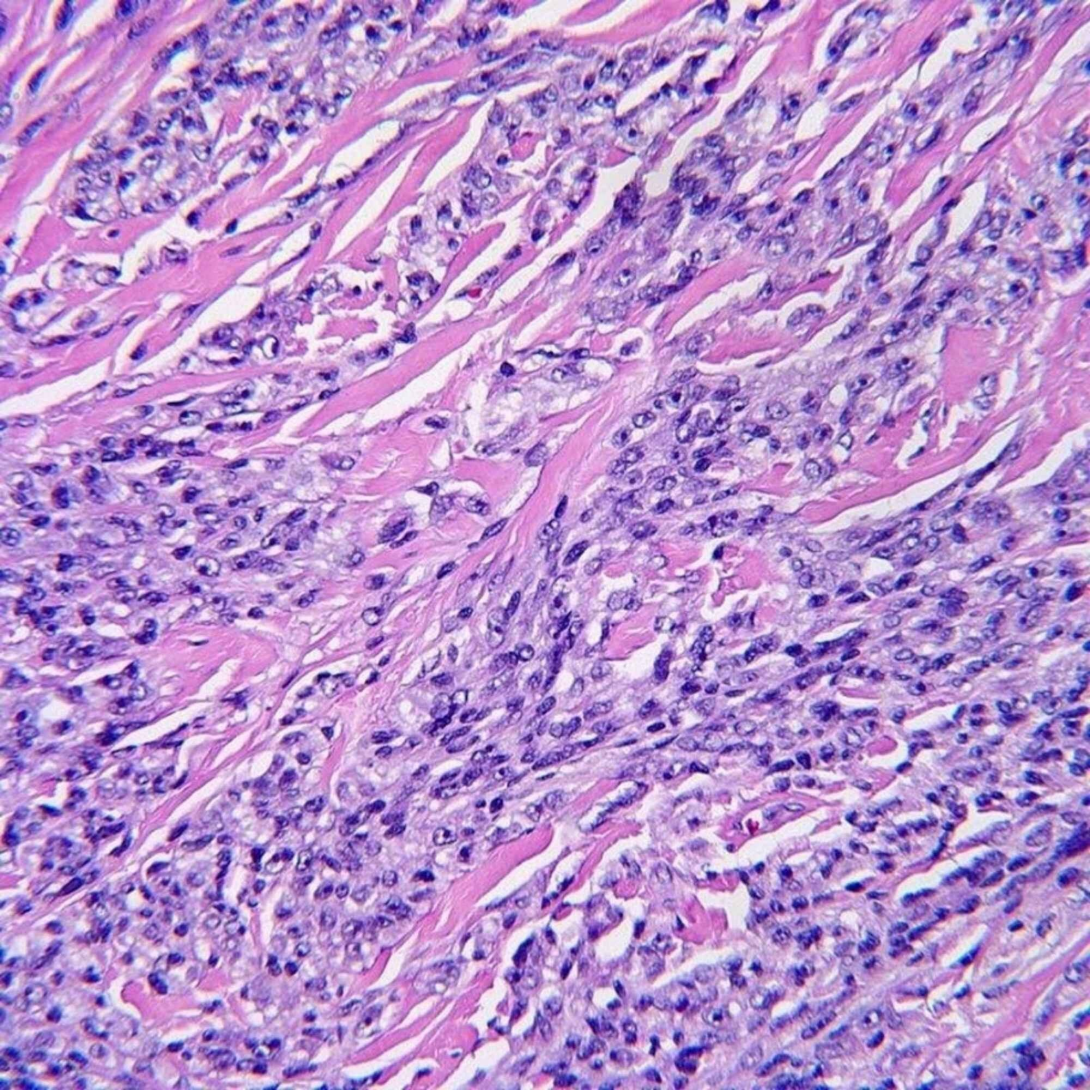
Histopathology. The lesional cells possess centrally located vesicular nuclei with prominent nucleoli.

**Figure 4:**
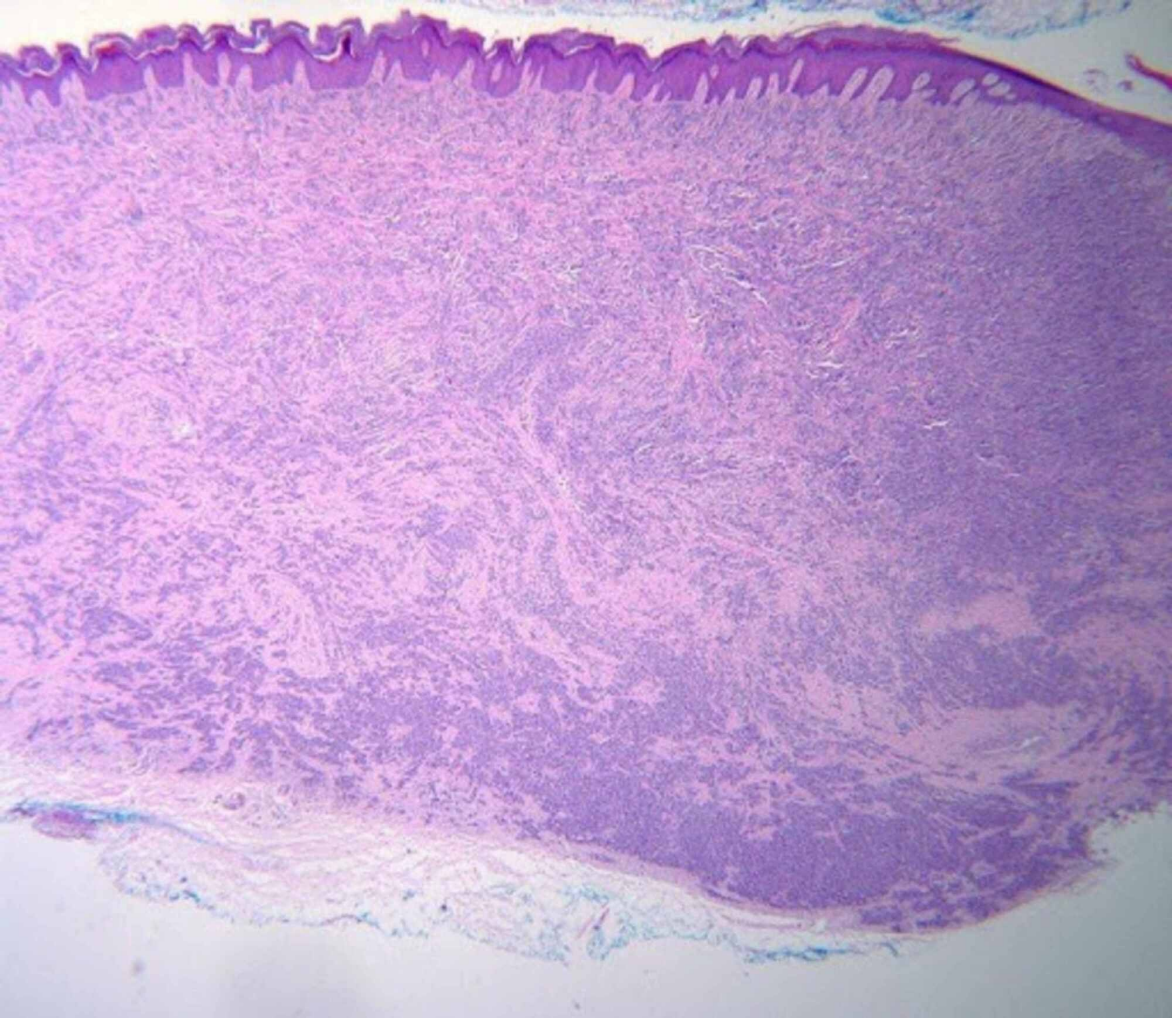
Histopathology. Histopathology revealed a well-circumscribed mass composed of nests and fascicles of uniformly fusiform cells throughout the dermis and into the subcutaneous tissue (H&E, original magnification x40).

**Figure 5:**
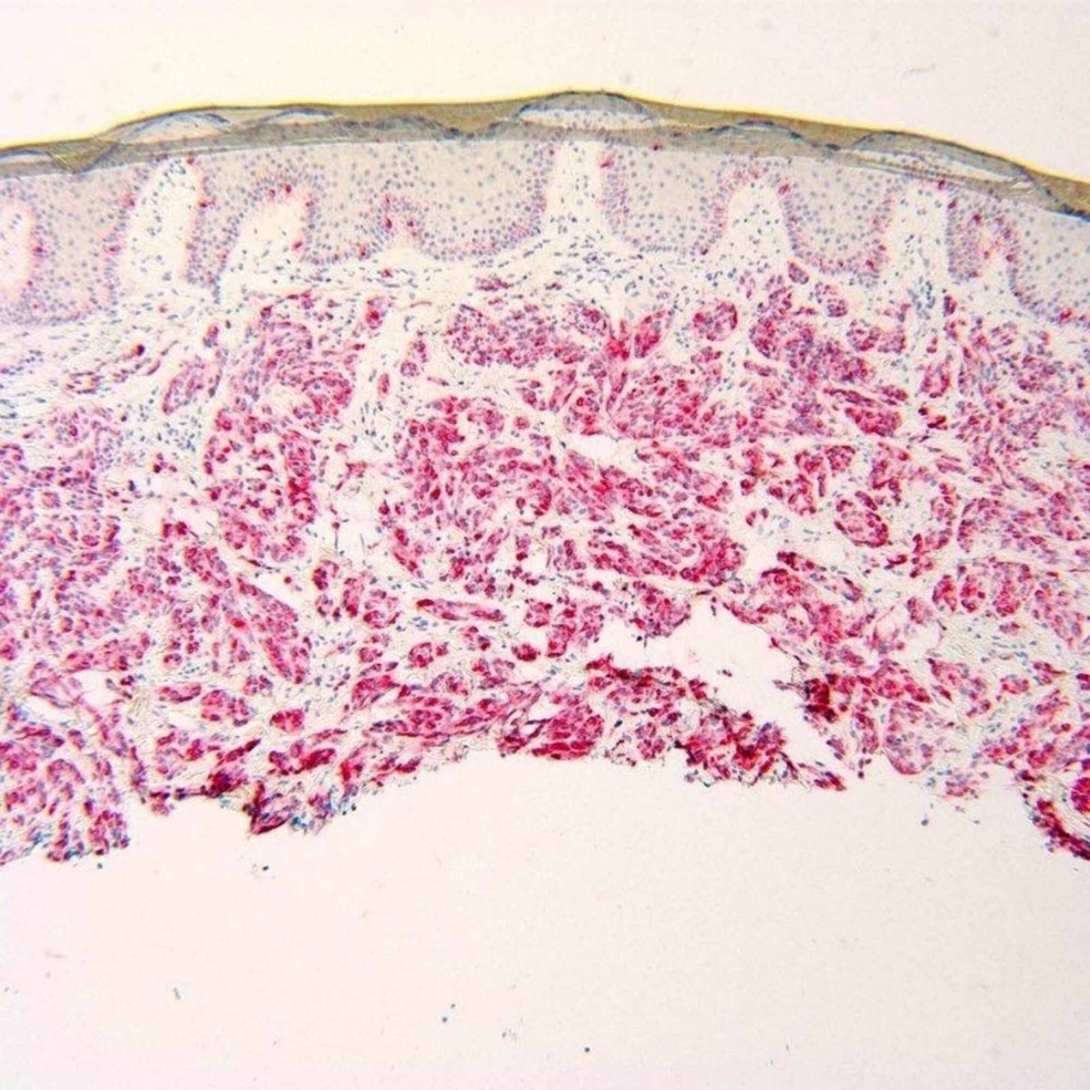
HMB-45 stain. Lesional cells staining positive in the dermis for HMB-45 immunoperoxidase. Note no epidermal involvement of the tumor.

Histopathologic examination of the lesion revealed infiltration of dermis and subcutis by an atypical spindle cell neoplasm composed of fusiform cells possessing ovoid nuclei with prominent nucleoli and amphophilic cytoplasm (Figures 2-3). Tumor cells were arranged in compact nests and short fascicles separated by a hyalinized stroma (Figure 4). The tumor cells showed diffusely positive cytoplasmic staining for HMB-45 (Figure 5), S100, and Melan A. Based on these findings, the diagnosis of CCS was considered.

**Figure 6:**
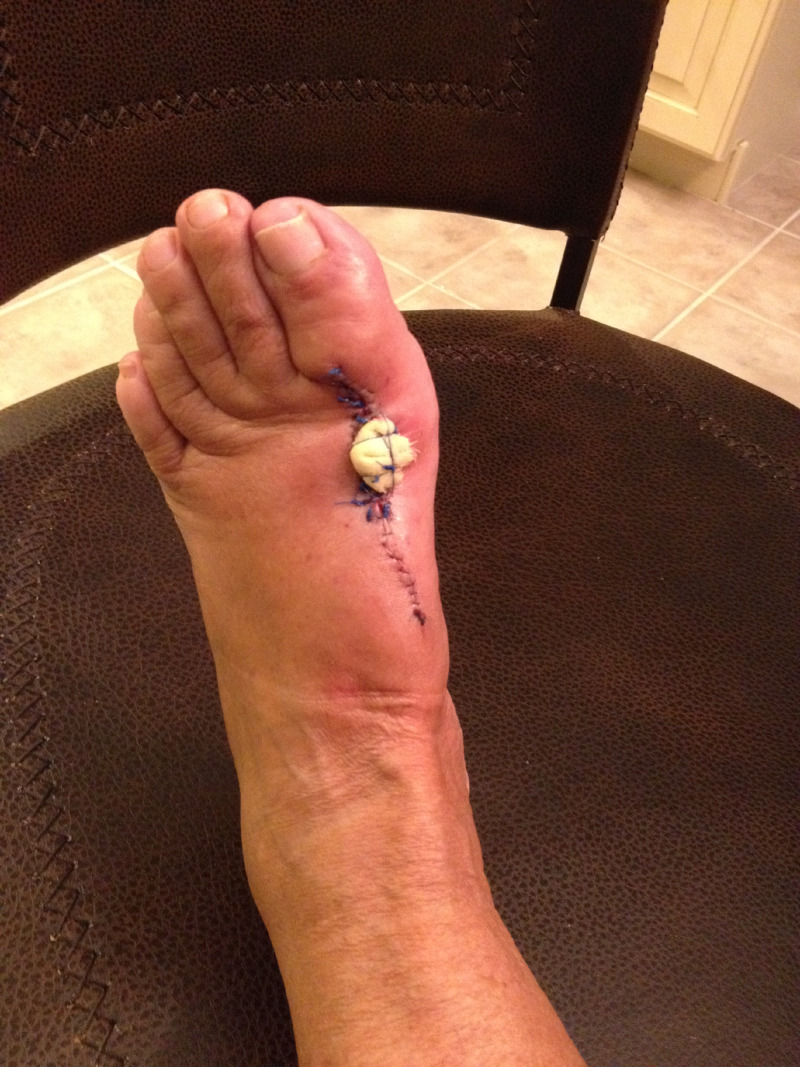
CCS postoperative. Post-wide excision of nodule with 1-cm margins. CCS, clear cell sarcoma

To confirm the diagnosis, a cytogenetic analysis was performed. A t(2;22)(q32;q12) chromosomal translocation resulting in a EWSR1/CREB1 fusion transcript was detected leading to a definitive diagnosis of CCS. For treatment purposes, the patient underwent a wide excision with 1-cm margins (Figure 6). Follow-up serologic evaluation and imaging with positron emission tomography-CT (PET-CT) revealed no metastatic disease. The patient continues to be closely monitored, and signs of metastatic disease remain negative.

## Discussion

Clear cell sarcoma is a malignant neoplasm most commonly involving the tendons and aponeuroses of the distal extremities. CCS is slightly more common in women than men. It typically occurs in young adults between the ages of 20 and 40 with a median age of 27 [1]. CCS shows little to no signs or symptoms in its primary stages. However, in its later stages, it can become aggressive and metastasize to the lymph nodes, lung, or bone [1]. The tumor is prone to local recurrence and widespread metastasis, usually many years after the diagnosis of the primary tumor. Therefore, close and consistent follow-up is recommended. The alternative term, “malignant melanoma of soft parts,” was described in 1983 secondary to the presence of cytoplasmic melanosomes within the tumor and histological similarities to malignant melanoma [1]. It may be difficult to differentiate malignant melanoma from CSS based on clinical presentation, histology, and immunohistochemistry alone, especially if the lesion is visibly pigmented [7].

Histologically, CCS displays small compact nests and fascicles of uniform to minimally pleomorphic fusiform-shaped or, less commonly, round tumor cells delineated by dense fibrous septa of tendons, fascia, and aponeuroses [5]. Superficial dermal lesions of CCS are rare. The neoplastic cells have a clear to pale-staining, eosinophilic cytoplasm with central round-to-ovoid vesicular nuclei containing prominent nucleoli. Mitotic activity is often low. In some instances, a variable number of multinucleated giant cells are present [6].

Immunohistochemically, CCS stains positive for S100, HMB-45, Melan A, MITF, and SOX 10 [2, 5]. This phenotype supports melanocytic differentiation of CCS and displays an identical histochemical profile with malignant melanoma. Diagnosis must be further reinforced by detection of chromosomal translocations unique to CCS. The major genetic distinction that differentiates CCS from malignant melanoma is its hallmark EWSR1 chromosomal translocation. In 75% of CCS cases, t(12;22)(q13;q12) results in an EWSR1/AFT1 fusion transcript [6, 8]. In a minority of cases, a t(12;22)(q32;q12) translocation results in the EWSR1/CREB1 fusion transcript [1]. Hisaoka et al. notes that only two of their 33 CCS specimens expressed the EWSR1/CREB1 fusion transcript after cytogenetic analysis [2].

This case highlights a rare presentation of CCS by older age of onset, arising in the superficial dermis, and containing the rare cytogenetic EWSR1/CREB1 fusion transcript. In addition, the longstanding clinical course of the lesion without metastasis is unique, as a majority of late-detected CCS cases result in eventual regional or distant metastases. With this case, we aim to bring awareness of this rare presentation of CCS so dermatologists may properly identify and manage this deadly cancer in an uncommon clinical setting. 

## Conclusions

We report an elderly patient with longstanding CCS on the left large toe, only developing tenderness in the area and seeking professional diagnosis after nearly 50 years. Our patient showed no signs of metastasis, which is rare after such a long course. This case serves as an example, as well as a reminder that CCS may have unique presentations and cytogenetic analysis, therefore requiring a high index of suspicion. Recognizing the possibility of CCS in an uncommon or unexpected setting may allow dermatologists to properly recognize, manage, and treat patients with CCS before metastasis occurs.
